# Influence of Ultraviolet and Oxygen Coupling Aging on Rheological Properties and Functional Group Index of Warm Mix Asphalt Binder

**DOI:** 10.3390/ma13194216

**Published:** 2020-09-23

**Authors:** Hailian Li, Peipei Tong, Xijun Zhang, Xixiong Lin, Bo Li

**Affiliations:** 1 National and Provincial Joint Engineering Laboratory of Road & Bridge Disaster Prevention and Control, Lanzhou Jiaotong University, Lanzhou 730070, China; lihailian@mail.lzjtu.cn (H.L.); zhangxijun1996@163.com (X.Z.); 2 Research Institute of Petrochina Fuel Oil Co. Ltd., Beijing 100195, China; tong-peipei@petrochina.com.cn; 3 PetroChia Karamay Petrochemical Co. Ltd., Karamay 834003, China; lhylxx@petrochina.com.cn

**Keywords:** warm mix asphalt, UV aging, oxygen aging, rheological properties, functional group index

## Abstract

Warm-mixed asphalt (WMA) has the characteristics of low mixing temperature and energy consumption, which makes it more suitable than hot-mixed asphalt for plateau regions (the altitude is above 500 m, and the terrain is relatively flat or has a wide area with certain undulations). However, WMA is subject to severe ultraviolet (UV) aging because the UV radiation in plateau areas is more intense. The asphalt’s aging changes its rheological properties inevitably, and thus degrades the asphalt pavement’s performance. Accordingly, the purpose of this paper is to investigate the effect of UV and oxygen coupling aging on WMA’s rheological properties and functional group index. Temperature and frequency sweep tests were performed with a dynamic shear rheometer. At the same time, the functional group index was used as an indicator to compare the changes in the WMA’s infrared spectrum before and after UV aging. The results showed that WMA’s elasticity increased and its viscosity decreased after aging with UV. Under the condition of UV–oxygen isolation, as the aging period increased, the WMA’s rutting factor increased gradually. The degree of improvement was greater than that under the condition of oxygen isolation. In contrast, the time required for the WMA’s complex shear modulus to recover under the condition of UV–oxygen coupling was delayed. An increase in the peak value of infrared spectrum indicates that the WMA has undergone oxygen aging. The presence and change in the carbonyl group reflects the degree of the WMA’s UV aging, and the UV aging condition had a greater effect on the peak values of the carbonyl and sulfoxide groups.

## 1. Introduction

Asphalt pavement has many advantages over cement concrete pavement such as flat surface, low noise and vibration, comfortable driving, a short construction period, and easy maintenance, so it is the form of pavement used most commonly for high-grade pavement in the highway industry. Asphalt is the main adhesive material in asphalt mixtures and is used widely in the fields of pavement construction because of its important properties including viscosity, viscoelasticity, and water resistance. As asphalt binder has higher viscosity, asphalt must be heated to a higher temperature in the production and construction processes. Typically, the range in the production temperature in the process of mixing is 150–160 °C and 170–180 °C for normal petroleum asphalts and polymer modified asphalts, respectively. As a result, a hot mix asphalt mixture consumes considerable energy in the process of mixing, transportation, paving, and compaction because of the high construction temperature. Furthermore, it emits a large amount of smoke and harmful gases that have adverse effects on construction workers and the surrounding environment. Therefore, innovations are necessary for cleaner production of hot asphalt mixtures to reduce energy consumption and environmental pollution.

Many energy-saving and environmentally-friendly technologies have been developed to solve these problems. Due to its advantages of low cost, environmental friendliness, and improved performance, WMA (Warm mix asphalt) has gradually become popular as an emerging green road construction technology [1,2]. This technology reduces viscosity to improve the ability to work asphalt binder in asphalt mixture construction by adding warm mix additives. Compared with the traditional hot mix asphalt mixture, WMA technology can reduce the mixing and compaction temperature of the asphalt mixture approximately 20–60 °C and does not sacrifice the asphalt mixture’s pavement performance. Zhang et al. found that different types of warming agents have significant effects on the road performance of asphalt mixtures. Organic additives can better improve their high temperature and water stability. Zeolites and emulsified asphalt warming agents can significantly improve their low temperature. For performance, a surfactant can improve its low temperature and water stability [3]. Zhou et al. found that a Sasobit warm mix can greatly improve the rutting resistance of the asphalt mixture, but reduce its water stability, low temperature crack resistance, and fatigue performance [4]. However, the reduced temperature of WMA also consumes far less fossil energy. Kanitpong found that, compared with traditional hot mix asphalt, WMA can reduce energy consumption by 18–30% [5]. In addition, WMA technology can reduce harmful greenhouse gas emissions effectively [6,7,8]. Due to their excellent characteristics, WMAs and mixtures have been extensively studied and applied in practice.

WMA makes it possible to construct highways in plateau regions, which has always been difficult to accomplish as these areas have low air pressure, low average annual air temperature, a fragile ecological environment, and short construction season. These can cause the hot mix asphalt mixture to cool rapidly, and thus, leads to insufficient compacting and inadequate performance [9]. Engineers try to produce HMA (Hot mix asphalt) asphalt mixtures at the appropriate mixing temperature and complete the construction before the minimum compaction temperature WMA technology allows. Using this measure can increase the construction temperature range of asphalt mixtures to compensate for the temperature loss in the mixture attributable to the low temperature environment and thus, the loss of asphalt mixture performance. Evidence has proven that this is a good method to construct highways in plateau areas, and some engineering projects in China have confirmed that WMA works well in construction in plateau regions using high temperature mixing and low temperature construction.

However, WMA in plateau regions experiences a more severe working environment, particularly strong UV (ultraviolet) radiation, which accelerates the aging of the surface material of the pavement, and shortens the pavement’s service life greatly [10,11]. When the asphalt mixture is applied to the road surface, it is exposed to sunlight during the service period, and undergoes a photo-aging reaction with UV rays [12]. UV aging caused changes in asphalt mixtures stiffness and ductility. Moreover, the effect of UV aging on the performance of asphalt pavement mainly occurred on the upper pavement. After this UV aging, the asphalt became brittle gradually, which caused the pavement to crack, seriously compromising its performance, and reducing its service life. Hence, aging behavior during asphalt service is considered a major factor that affects the asphalt pavement’s durability [13,14]. However, because of many factors, UV aging research is very complex, time consuming, and difficult [15]. Thus, aging simulations in the laboratory are still one of the most effective methods to study the UV aging properties of asphalt.

At the same time, as a viscoelastic material, WMA’s performance is related closely to its rheological properties [16]. Therefore, it is necessary to study the effect of aging on these properties. Xiao et al. studied WMA’s rheological properties after thermal aging, in addition, creep recovery, amplitude, and frequency sweep tests showed that the binders with Sasobit had a slightly higher complex modulus, but exhibited lower creep compliance and phase angle than the binder containing other WMA additives, regardless of asphalt type and grade. However, they did not conduct a detailed study of its UV aging characteristics [17]. Shenoy used the accelerated test to simulate the aging process in the field, and rheological characterization of the aged asphalt to measure the degree of hardening attributable to aging. The high temperature rheological properties of aged asphalt were analyzed [18], and the results depended on the relation between the displacement factor of the asphalt’s main curve before and after aging. Through a correlating equation for the rheological property, it was shown that estimates of the high temperature dynamic rheological properties of the aged asphalt could be obtained without actually aging the binder in the laboratory [19,20]. By comparing the asphalt’s physical and rheological properties before and after UV aging, Zeng et al. concluded that temperature had no significant effect on the asphalt’s UV aging resistance at temperatures below 50 °C [21]. Wang et al. studied the influence of aging on the rheological properties of SBS (Styrene-Butadiene-Styrene) modified asphalt and rubber asphalt, respectively, and the results showed that the effect of aging on SBS modified asphalt was greater than that of CR (Crumb Rubber) modified asphalt [22,23], while Zhu studied the aging properties of original and modified asphalt as well as the glass transition, modulus, and viscoelastic composition of asphalt at low temperature [24]. Rojas and Huang, respectively, studied the relation between polymer-modified asphalt and SBS modified asphalt’s rheological properties and microstructure [25,26], and Xiao et al. analyzed and studied the influence of aging on CR modified asphalt’s structure from the micro perspective [27]. However, there are few studies on the effects of UV–oxygen coupled aging conditions on WMA’s rheological properties. Therefore, it is of great significance and engineering application value to study these properties of WMA in a high altitude environment, particularly under strong UV radiation.

Some researchers have also conducted long-term, extensive research on asphalt’s UV aging in plateau areas, and their primary results have shown that, during the pavement’s actual use, the asphalt’s oxidative aging occurs initially on the surface of the pavement [28]. Under the combined action of UV light and atmospheric oxygen, the asphalt on the road surface hardens gradually and becomes more prone to cracks. Subsequently, the oxygen penetrates downward gradually, and oxidizes the pavement’s asphalt continuously. Existing research has shown that atmospheric oxygen can penetrate 15 mm below the surface [29]. Corbett and Mertz studied the four component changes after asphalt’s long-term aging, and their results showed that the saturates of asphalt did not change significantly after long-term aging [30]. During the asphalt’s aging process, oxygen-bearing functional groups form in the asphalt molecule, which transfer aromatics and colloids in the asphalt to the asphaltenes [31]. Wu et al. found that a large amount of UV degradation occurred during UV aging, during this process, the asphalt was oxidized [32]. This indicates that UV light and oxidation has a great effect on the asphalt’s performance. However, current research has not distinguished between the effects of oxygen and light on aging and its rheology, which is necessary to help prevent WMA from aging.

The purpose of this study was to study the effects of different UV–oxygen coupling aging conditions on the rheological properties of WMA to obtain its rheological properties under UV aging, and to improve its anti-UV aging. WMA was prepared by adding Evotherm M1 (Meadwestvaco, Richmond, VA, USA) warm mix agent to the original asphalt. First, the WMA was aged by RTFOT (Rolling Thin Film Oven Test), and then subjected to UV aging for 50 h using a custom-made UV aging tank. Temperature and frequency sweep tests were carried out with a DSR (Dynamic Shear Rheometer) to obtain the complex modulus, phase angle, and rutting factor of WMA aged to different degrees.

## 2. Materials and Methods

### 2.1. Materials

The original asphalt is SK90# asphalt. The asphalt’s basic performance test was conducted in accordance with the Chinese standard “Highway engineering asphalt and asphalt mixture test regulations” (JTG E20-2011) [33]. Its performance indicators are shown in Table 1, and were consistent with the requirements of China’s “Technical Specifications for Construction of Highway Asphalt Pavements” (JTG F40-2004).

The warming agent selected was Evotherm M1 with representative organic viscosity reduction and surface temperature mixing mechanism types. Evotherm M1 is a low viscosity liquid chemical additive based on the emulsified asphalt viscosity reduction technology produced by Mead Westvaco, as shown in Table 2.

### 2.2. Experimental Plan

#### 2.2.1. Preparation of Warm Mix Asphalt

**Figure 1:**
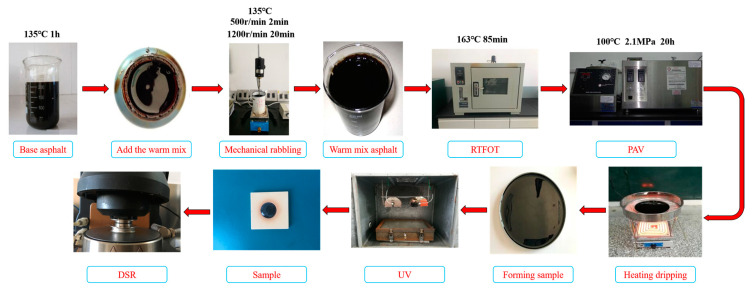
Flow chart of WMA preparation and test.

Evotherm M1 WMA was prepared in this study with a blending amount of 0.5% of the mass of the asphalt. In addition, to avoid errors attributable to different preparation processes, and ensure the full fusion of the warming agent with the original asphalt, the Evotherm M1 warming agent’s preparation method was as follows:

First, the original asphalt as heated to a flowing state in an oven at 135 °C, and then an appropriate amount of hot asphalt was poured into a preparation vessel. Next, the asphalt was stirred at low speed at 135 °C with a mechanical stirrer for 2 min, and Evotherm M1 was added while stirring. Finally, the mixture was stirred at 1200 rpm for approximately 20 min to make the Evotherm WMA required. The preparation process is shown in Figure 1. As the stirring process is lengthy, it should be heated properly while stirring the WMA mechanically, but the temperature should not be too high to prevent the asphalt from aging during the preparation process. Due to the small mixing proportion of Evotherm M1, to facilitate operation and prevent “over-mixing”, it is recommended to use a syringe as an auxiliary device to add the Evotherm M1 warm mixing agent.

#### 2.2.2. Preparation of Short-Term Aging Asphalt Samples

The Rotating Thin Film Oven Test (RTFOT) is used largely to simulate asphalt’s short-term thermal oxidative aging. It can simulate the mixing and paving aging process of asphalt accurately, and the asphalt can be characterized conveniently after aging to analyze the aging process and changes in the asphalt’s performance. In this paper, the RTFOT test was carried out according to the method specified in the Test Procedure for Highway Engineering Asphalt and Asphalt Mixture (JTG E20-2011), in which the aging temperature is 163 °C ± 0.5 °C, the air flow rate is (4000 ± 200) mL/min, and the aging time is 85 min [33].

#### 2.2.3. Preparation of Ultraviolet Aged Asphalt Samples

**Figure 2:**
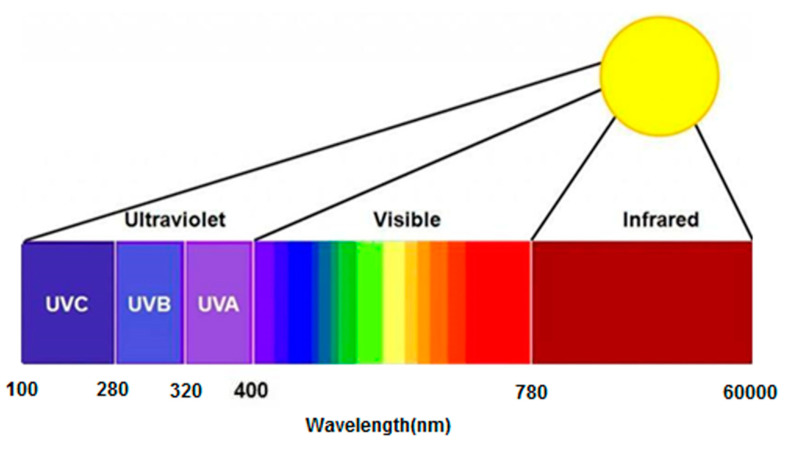
Sun spectrum distribution.

According to the size of the wavelength, the solar spectrum can be divided into ultraviolet light (100–380 nm), visible light (380–780 nm), near infrared (780–2500 nm), and far infrared (2500–60,000 nm). Among them, the wavelength of 100~280 nm is UV-C, 280~320 nm is UV-B, and 320~380 nm is UV-A (as shown in Figure 2) [21]. The UV-C wavelength between 100 nm and 280 nm is the shortest strong wave. Although it is harmful to biology, it cannot reach the ground due to the strong absorption of ozone, which causes no harm to the asphalt pavement. UV-B, with a wavelength of 280~320 nm, belongs to the medium wave with strong effect, which is more harmful to biology. Most of them are absorbed by ozone and reach the ground relatively less. In the solar spectrum, in the 320~380 nm wavelength, UV-A has the highest energy, cannot be completely absorbed by ozone, reaches the ground of UV-A, and UV-B accounts for 5% of the solar spectrum, but as a result, UV-A and UV-B can destroy the molecular structure of asphalt materials, leading to asphalt pavement performance decay, thus simulating the natural conditions of ultraviolet aging requires choosing UV-A as a UV light source.

**Figure 3:**
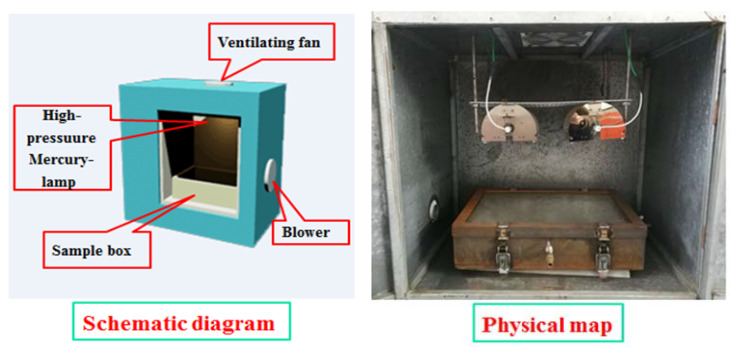
UV and oxygen coupling aging simulation box.

Heat, oxygen, light, and other environmental factors affect asphalt pavement and its durability during the pavement service lifetime. To study the UV aging of WMA under the conditions of oxygen isolation and UV–oxygen coupling, a sample simulation box placed under UV aging conditions was designed to perform the UV aging test of asphalt samples under oxygen isolation. The self-designed UV aging simulation system consists of two parts. The first part is the UV aging case, which included a UV aging box, air cooling system, UV lamp set, and temperature monitoring system. A high-pressure mercury lamp (model GYL230-220V-1000W, Noerka, Ningbo, China) was placed in the indoor UV aging environment box 23 cm from the asphalt sample, and the irradiation intensity was 200 ± 2 w/m^2^. The second part is the ultraviolet aging sample part, which included the UV aging sample box and gas exchange system (Figure 3). A weight of 50 ± 0.5 g of asphalt was placed into the asphalt aging tray with a diameter of 14 cm. The specific operation method was as follows: the asphalt sample was placed in the sample box and sealed, the air inlet and exhaust port were opened, and nitrogen was injected to discharge oxygen. The oxygen concentration in the sample box was verified by the ignition test and closed the inlet and exhaust ports after the gas exchange.

Relevant studies have shown that UV light is the primary cause of the aging of asphalt pavement within the first four months after the pavement is laid [34]. Therefore, the indoor UV test in this paper simulated natural outdoor aging for four months. With reference to the annual solar radiation in western Inner Mongolia, the maximum radiation amount of 7000 MJ/m^2^ was selected as the total amount of simulated annual solar radiation, and the annual average value of ultraviolet radiation in its total radiation is about 6%, that is, the simulation of the total annual ultraviolet radiation was 420 MJ/m^2^ when the asphalt sample was 23 cm away from the high-pressure mercury lamp, the temperature of the sample surface was stable at 68 ± 3 °C, and the irradiation intensity was stable at 200 ± 2 w/m^2^. According to Equation (1), the annual UV exposure time simulated was 532.7 h, and the 4-month UV exposure time was 177.6 h, according to the relevant conversion formula. This test can be carried out conveniently according to these UV aging rules, and the indoor UV aging tests were performed at five durations: 0, 50, 100, 150, and 200 h.

Indoor simulated outdoor ultraviolet radiation for one year.

(1) $$\frac{{420\;\mathrm{MJ}/m}^{2}}{{200\;W/m}^{2}}\approx 583\;h$$

#### 2.2.4. Rheological Property Tests

Asphalt can be regarded as a viscoelastic material from a mechanical point of view, and temperature and stress affect its rheology. The poor rheological properties of asphalt lead to ruts, fracture, and other distresses that reduce the asphalt pavement’s service life seriously. The United States SHRP (Strategic Highways Research Program) plans to use a dynamic shear rheometer (DSR) to measure the complex shear modulus, G*, and phase angle, δ, at a specified angular velocity (ω is 10 rad/s) and temperature of an asphalt sample sandwiched between the shock plate and the fixed plate to evaluate the asphalt binder’s viscoelastic properties. The Superpave specification uses the rut factor G*/sin δ to characterize the binder’s high-temperature resistance to permanent deformation, and measures the asphalt’s high-temperature rut resistance. The smaller the G*/sin δ, the lower the high temperature performance. G* is the ratio of total shear stress to total shear strain, which reflects the stiffness modulus of the material against deformation, and consists of two parts: elastic modulus G′ (recoverable part) and viscous modulus G″ (non-recoverable part). The phase angle, δ, is the time lag between the asphalt’s stress and strain in the test state, and is the ratio of the elastic and viscous components. For a completely elastic material, the phase angle is approximately zero, and for a completely viscous material, the phase angle is near 90° [35].

In this paper, TA Instrument’s AR 1500ex rheometer (New Castle, DE, USA) was used to measure the temperature or frequency complex shear modulus, G*, and phase angle, δ, of WMA under different UV aging conditions to analyze the influence of UV aging on the WMA’s rheological properties. The temperature sweep range of the asphalt was 52~82 °C, the temperature range was 6 °C, the target strain was 1% [35,36], and the angular frequency was 10 rad/s. The frequency sweep range was 0.1~100 rad/s, and the asphalt’s test temperature was controlled at 58, 70, 76, and 82 °C. In this experiment, we prepared three replicates of asphalt for each test, and set three parallel tests and guaranteed the error of three parallel tests before the average value was taken.

#### 2.2.5. Fourier Transform Infrared (FTIR) Test

The Thermo Fisher Nicolet Fourier transform attenuated total reflection infrared spectrometer (Thermo Scientific™, Waltham, MA, USA) was used to perform the infrared spectroscopy test, and samples of WMA under different aging conditions were applied to ZnSe crystal plates. The primary test parameters were: scan range = 400~4000 cm^−1^; number of scans = 32, and minimum resolution = 0.019 cm^−1^.

## 3. Results and Analysis

### 3.1. Temperature Sweep Test

#### 3.1.1. Complex Shear Modulus Curve

**Figure 4:**
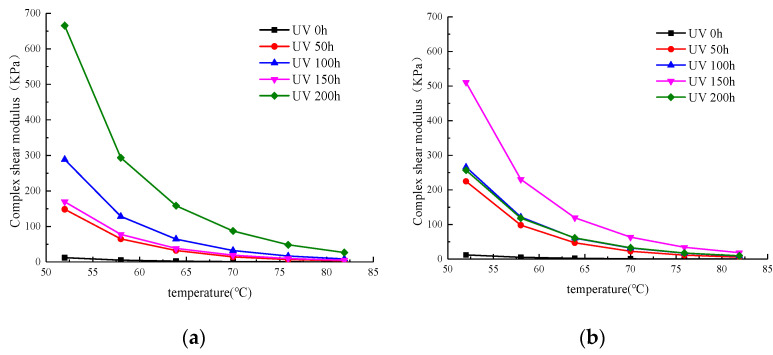
Complex shear modulus of WMA. (**a**) Oxygen isolation conditions. (**b**) Oxygen-containing conditions.

The complex shear modulus curves of WMA with different aging times are shown in Figure 4a. As the figure shows, as the temperature rose, the complex shear modulus always showed a downward trend overall, and the maximum degree of decline was in the range of 52 to 58 °C. As the temperature increased, the declining trend slowed gradually. At the same temperature, as the aging time increased, the complex shear modulus also increased. When the aging time increased to 150 h, the complex shear modulus became smaller, and was very close to the curve at 50 h. As the aging time continued to increase, when it reached 200 h, the complex shear modulus had increased significantly. These results show that UV aging has a great influence on the total resistance of asphalt under repeated shear deformation, and the influence on the complex shear modulus varies as the UV aging time increases.

From Figure 4b, we concluded that when the aging time was 0, 50, 100, and 150 h, the SK (Asphalt produced by SK, Soul, Korea) WMA’s complex shear modulus increased as the UV aging time increased. However, when the aging time was extended to 200 h, the complex shear modulus decreased instead, which was similar to the complex shear modulus curve when the UV aging time was 100 h. This differed from the WMA’s complex shear modulus after aging at different times in the oxygen-suppressing condition shown in Figure 3, where the complex shear modulus decreased after 150 h of aging. The results show that under the condition of oxygen isolation, UV aging has a great influence on the causes of asphalt’s total resistance repeated shear deformation; when the aging time is increased to 150 h, the change law of complex shear modulus is not clear, but it can be seen that the time at which the SK WMA’s complex shear modulus is restored under the condition of UV–oxygen coupling being delayed.

#### 3.1.2. Complex Shear Modulus (70 °C)

**Figure 5:**
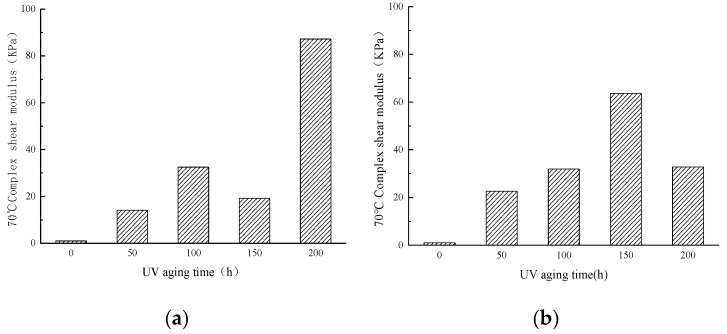
Complex shear modulus (70 °C) of WMA. (**a**) Oxygen isolation conditions. (**b**) Oxygen-containing conditions.

Figure 5a shows the complex shear modulus at 70 °C measured by adding 0.5% Evotherm SK WMA under oxygen isolation conditions after UV aging for different times. Figure 5a illustrates that SK WMA’s complex shear modulus after short-term aging at 70 °C was 1.00 KPa, and after 50 and 100 h UV aging, it increased to 14.09 KPa and 32.47 KPa, respectively. However, when the SK WMA UV aging time was 150 h, the complex shear modulus at 70 °C decreased to 19.18 KPa, which is only 5.09 KPa different from that of the UV aging at 50 h. When the aging time was 200 h, the complex shear modulus at 70 °C increased greatly to 87.22 KPa.

Figure 5b shows the complex shear modulus at 70 °C measured after UV aging at different times under aerobic conditions with SK WMA that contained 0.5% Evotherm. It can be seen from Figure 5b that the complex shear modulus was 1.00 KPa after short-term aging of SK WMA at 70 °C, and after UV aging for 50, 100, and 150 h, it increased to 22.64 KPa, 31.92 KPa, and 63.59 KPa, respectively. However, when the SK WMA’s UV aging time at 70 °C was 200 h, the complex shear modulus decreased to 32.73 KPa, which was only 0.81 KPa different from that of UV aging at 70 °C for 100 h.

#### 3.1.3. Phase Angle Curve

**Figure 6:**
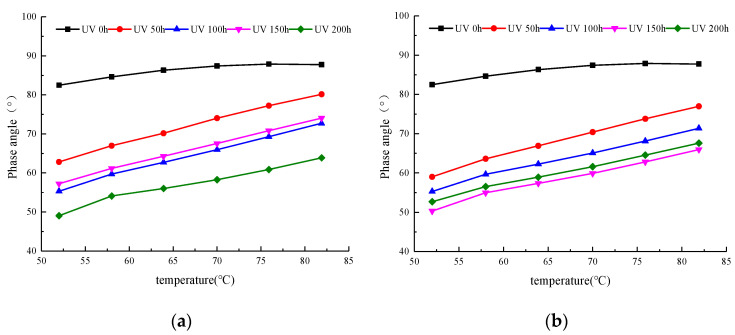
Phase angle of WMA. (**a**) Oxygen isolation conditions. (**b**) Oxygen-containing conditions.

The phase angle curves of WMA with different aging times are shown in Figure 6a. When SK WMA was not aged with UV, its phase angle maintained a small change in the range of 80–90 °C as the temperature increased, and it always remained in a viscous flow state. After UV aging, the phase angle increased at a faster rate as the temperature increased. When the temperature remained constant, the WMA’s phase angle tended to decrease initially, and then increased and decreased with an increase in the aging time. These results demonstrate that SK WMA’s elasticity increases, while its viscosity decreases after UV aging.

The curve in Figure 6b showed that after the SK WMA’s UV aging, its phase angle decreased greatly, and, as the temperature increased, the phase angle’s rate of increase was higher than without UV aging. When the aging time was 50, 100, and 150 h, the SK WMA’s phase angle decreased, while it increased slightly when the aging time was 200 h. Furthermore, Figure 6b shows that after UV aging for different times, the maximum phase angle value was less than that without UV aging. This confirms that UV aging has a great influence on the SK WMA’s total resistance when it is subjected to repeated shear deformation. Similar conclusions to complex shear modulus, the change law of phase angle with the increase of aging time was not obvious. It is worth noting that when the aging time reached 150 h, the viscoelastic ratio of asphalt changed significantly.

#### 3.1.4. Rutting Factor Curve

**Figure 7:**
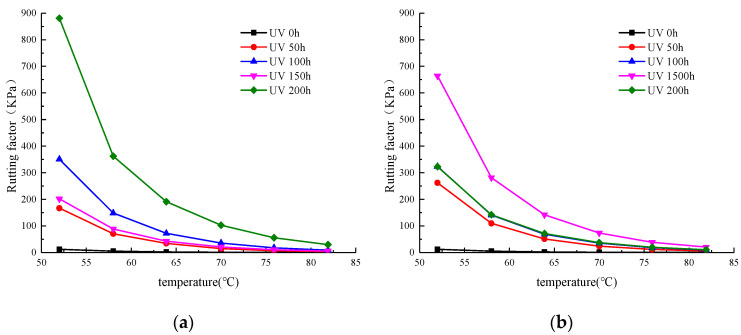
Rutting factor of WMA. (**a**) Oxygen isolation conditions. (**b**) Oxygen-containing conditions.

The rutting factor (G*/sinδ) is used to characterize the ability of asphalt materials to resist rutting deformation under high temperature conditions, and the larger the G*/sinδ, the better the high temperature deformation resistance of the material, so the basic assumption is that a stiffer binder can provide a good rut resistant potential. Figure 7a shows a temperature sweep curve of the rutting factor at various temperatures after UV aging at different times under oxygen shielding conditions. It can be seen from the figure that as the temperature increased, the rutting factor reduced gradually, and the rate of decay also declined. Similarly, when the temperature was held constant, the rutting factor increased with aging time. When the aging time increased to 150 h, the rutting factor decreased to a value similar to that at the aging time of 50 h. This is similar to the change trend in the WMA’s complex shear modulus with different aging times, as shown in Figure 4. The above results show that UV aging has a significant effect on rutting performance.

Figure 7b is a temperature-sweep curve of the rutting factor of SK WMA with 0.5% Evotherm at various temperatures after UV aging at different times under aerobic conditions. As the figure shows, the rutting factor increased with the aging time within 150 h. However, with the aging time of 200 h, the rutting factor declined, and its curve was similar to that when the UV aging time was 100 h. This differed significantly from the rutting factor after aging at different times under the oxygen isolation condition. Although the rutting factor was larger under the oxygen isolation condition than under the aerobic conditions with UV aging for 200 h, it was slightly lower under the oxygen isolation condition than under aerobic conditions within 100 h of UV aging.

### 3.2. Frequency Sweep Test

#### 3.2.1. Main Curve of Complex Shear Modulus

**Figure 8:**
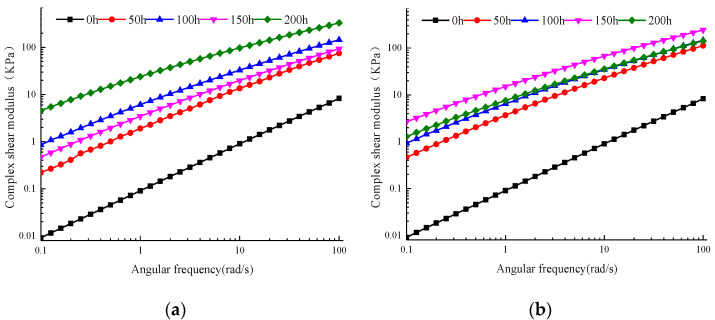
Main curve of complex shear modulus of WMA (70 °C). (**a**) Oxygen isolation conditions. (**b**) Oxygen-containing conditions.

Figure 8a shows the frequency sweep curve of the WMA’s complex shear modulus after aging for different times. As can be seen in the figure, as the load frequency increased, the complex shear modulus always increased at a rapid rate. At a certain frequency, when the aging time was less than 100 h, the complex shear modulus always increased as the aging time increased. When the aging time increased from 100 to 150 h, the complex shear modulus decayed, and its value was between the corresponding values of the aging time of 50 and 100 h. As the aging time continued to increase, the complex shear modulus increased again. These results show that in the process of increasing the load frequency when the ambient temperature remains constant, the total resistance the SK WMA generates under repeated shear deformation tends to increase on the whole, but also decreases in the process.

As can be seen in Figure 8b, the SK WMA’s complex shear modulus increased greatly up to 50 h of UV aging. When the aging period was extended to 100–150 h, its range was small, while when the aging was 200 h, it decreased slightly. These results indicate that at the same ambient temperature, the SK WMA’s total resistance under repeated shear deformation increased as the UV aging increased. However, the SK WMA’s total resistance under repeated shear deformation declined gradually at 150–200 h of UV aging. By comparing this figure with Figure 8a, it can be seen that the complex shear modulus under the UV–oxygen coupling condition after UV aging for 50–150 h was relatively slightly larger than that under the UV–oxygen coupling condition. When the UV aging period was 200 h, the complex shear modulus decreased slightly under the UV–oxygen coupling conditions, but increased sharply under the oxygen-blocking condition.

#### 3.2.2. Main Curve of Phase Angle

**Figure 9:**
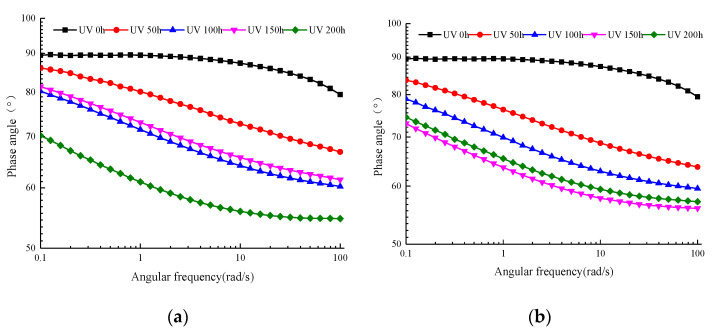
Main curve of phase angle of WMA (70 °C). (**a**) Oxygen isolation conditions. (**b**) Oxygen-containing conditions.

As Figure 9a shows clearly, the SK WMA’s phase angle after UV aging for different times decreased gradually as the angular frequency increased. However, because the aging time increased, the phase angle’s rate of change changed significantly as the angular frequency increased. Before UV aging, the SK WMA’s angular frequency first decreased slowly in the low frequency range as the angular frequency increased, and the phase angle decreased until the high frequency range. After aging SK WMA, as the aging time increased, the area where the phase angle was reduced greatly shifted gradually to the low frequency range, and the phase angle’s range decreased in the high frequency range. The SK WMA’s phase angle increased greatly within 50 h of UV aging, and continued to increase when the aging time was extended to 100 h, and then decreased when the aging time was 150 h. When the aging time increased to 200 h, the phase angle increased greatly.

Figure 9b shows that after SK WMA was aged with UV, as the UV aging period increased, the region where the phase angle was reduced greatly shifted gradually toward the low frequency range, and the range of the phase angle range decreased in the high frequency range. This is similar to the change trend in the phase angle after UV aging under the condition of oxygen isolation shown in Figure 9a. The SK WMA’s phase angle increased greatly within 50 h of UV aging, and continued to increase when the aging time was extended to 150 h, while it decreased when the aging period was 200 h. This indicates that UV aging under the condition of UV–oxygen coupling causes SK WMA’s elasticity to increase and its viscosity to decrease.

### 3.3. FTIR Analysis

**Figure 10:**
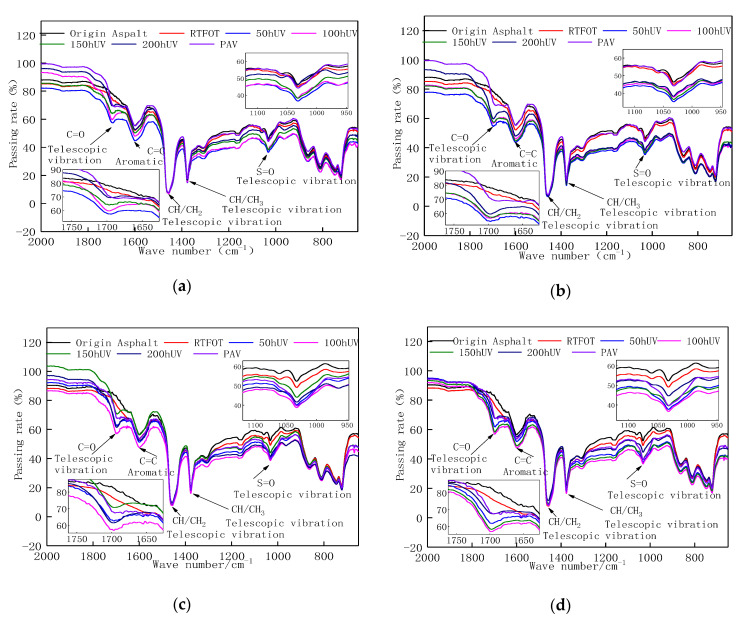
WMA’s infrared spectra before and after aging. (**a**) 0% E Isolation oxygen conditions. (**b**) 0% E oxygen-containing conditions. (**c**) 0.5% E Isolation oxygen conditions. (**d**) 0.5% E oxygen-containing conditions.

According to the change rule of Evotherm M1 WMA’s rheological properties after aging, RTFOT aging, UV aging, and PAV (pressurized aging vessel) aging, can all reduce WMA’s rheological properties. This is attributable primarily to WMA’s photo–thermal–oxygen coupling aging, which alters its microscopic composition. To clarify the changes in WMA’s microscopic composition after UV aging, a Fourier transform attenuated total reflection infrared spectrometer was used to analyze the Evotherm M1 WMA’s infrared spectrum before and after UV aging to study the change in functional groups. The infrared spectra of WMA with different dosages of Evotherm M1 before and after aging are shown in Figure 10.

It can be seen from Figure 10 that during the Evotherm M1 WMA’s UV aging process, the highest value of the carbonyl (C=O) absorption peak at 1700 cm^−1^ and the sulfoxide (S=O) expansion vibration peak at 1030 cm^−1^ increased significantly. Comparing the effects of the two UV aging conditions, oxygen-isolation and UV–oxygen coupling, on the highest values of the absorption peaks of the carbonyl and sulfoxide groups in Evotherm WMA, it was found that UV–oxygen coupling aging conditions had a greater effect on the highest values of the two absorption peaks. Figure 10 also shows a comparison between the infrared spectra of the two amounts of Evotherm M1 mixed in the WMA after UV–oxygen coupling aging and the results of the original, RTFOT aging, UV aging, and PAV aging. It was found that carbonyl (C=O) characteristic functional groups appeared generally at 1700 cm^−1^ after UV–oxygen coupling aging and PAV aging, and the peak value and area of this functional group increased as the aging time was extended.

We calculated the carbonyl (*I_CO_*) and sulfoxide index (*I_SO_*) of all asphalt samples separately. The tangent line of the lowest point on both sides of the absorption peak was used as the calibration baseline to calculate the absorption peak area, and the FTIR spectrum of the asphalt cement was quantitatively analyzed according to Lambert–Beer law [37]. The detailed results of the calculations are shown in Table 3.

From Table 3, we can see that compared with the oxygen isolation condition, it was found that the UV–oxygen coupling aging had a more significant effect on the area and the increase in the peak of the carbonyl (C=O) characteristic functional groups. The change in functional groups after PAV aging was similar to that after 50 h of UV aging. The peak carbonyl group of the original asphalt and Evotherm M1 WMA tended to be stable after 150 h of UV aging. It can be seen from the data that carbonyl is the formation is the mark of carbonyl acid or ketone, and is the characteristic peak of asphalt aging. The presence and change in the carbonyl group reflected the degree of the asphalt’s UV aging. As the UV aging time increased, and with the participation of oxygen, the characteristic peaks of the carbonyl groups increased continuously, indicating that UV radiation and oxygen accelerate the process of asphalt’s UV–oxygen coupling aging. After addition of the WMA additive, the results at 150 °C showed similar trends as with the rheological measurements. This shows that after 150 h of UV aging, the WMA was basically completely aging. After RTFOT was aged for 85 min, the peak value of the sulfoxide (S=O) functional group at 1030 cm^−1^ increased slightly, indicating that the sulfoxide had undergone a slight thermal oxygen aging under short-term thermal oxygen conditions. The sulfoxide group has a peak that characterizes the asphalt’s oxygen aging, and the increase in the peak value indicated that the asphalt had undergone oxygen aging.

## 4. Conclusions

To investigate the effect of UV aging conditions on WMA’s rheological properties, UV–oxygen coupling aging and oxygen-isolation UV aging were used in this research to simulate the aging of WMA. After UV–oxygen coupling aging or oxygen-isolation UV aging, the complex shear modulus, rutting factor, phase angle, and the area and peak value of the WMA’s characteristic functional group were investigated. The following conclusions were drawn from this study:

(1) The UV aging period had a significant effect on the total resistance of the asphalt to repeated shear deformation causes. As the UV aging time increased, its influence on the complex shear modulus rose generally. Under the condition of oxygen-isolation, UV aging’s effect on WMA’s total resistance generated by repeated shear deformation was greater than that of UV–oxygen coupling.
(2) UV aging had a great influence on WMA’s viscoelasticity, which increased as the UV aging time increased in a certain period. UV radiation coupling effects can accelerate the oxidation rate. When the ultraviolet aging reaches a certain time, the influence of oxygen reduces the high temperature stability of asphalt.
(3) The film formed on the surface of the WMA during the UV aging process hindered the aging process, and under the condition of light–oxygen coupling aging, UV was more destructive to the film, which accelerated the rate at which UV penetrated into the asphalt.
(4) WMA’s rheological properties changed after UV aging primarily because aging changed the peak value of the carbonyl (C=O) functional group. Furthermore, oxygen had a significant influence on the increase in the area and peak value of the characteristic carbonyl functional group.
(5) Some conclusions obtained now do not explain well the unconventional laws that occur after 150 h of UV aging. These conclusions are only limited to the equipment used in this study and they may be different for other equipment.

## Figures and Tables

**Table 1:** Physical properties of the asphalt binder.

Tests		Units	SK90#
Penetration (25 °C, 100 g, 5 s)		0.1 mm	91.2
Penetration Index PI		-	−1.48
Ductility (5 cm/min, 10 °C)		cm	>100
Wax content (distillation method)		%	2.0
Softening point (°C)		°C	45.8
60 °C dynamic viscosity		Pa·s	145
Rotating thin film oven test (RTFOT) (163 °C, 85 min)	Loss of quantity/%	%	66
	Penetration ratio/%	%	0.06
	Ductility (5 cm/min, 10 °C)	cm	9.1

**Table 2:** Properties of warm mix additives.

Items	Exterior	Viscosity/(Pa·s)	Density/(g/cm^3^)	Proportion/%
Evotherm M1	Yellow brown sticky liquid	950 (± 50) × 106	0.97 (± 0.05)	Quantity of asphalt 0.6

**Table 3:** WMA’s *I_CO_* and *I_SO_* before and after aging.

Asphalt Type	Characteristic Peak Index	Origin Asphalt	RTFOT	PAV	50 h UV	100 h UV	150 h UV	200 h UV
(a)	*I_CO_*	0.078	0.103	0.181	0.197	0.203	0.304	0.310
	*I_SO_*	0.178	0.182	0.257	0.274	0.286	0.305	0.323
(b)	*I_CO_*	0.078	0.103	0.286	0.304	0.313	0.373	0.381
	*I_SO_*	0.178	0.182	0.191	0.205	0.233	0.257	0.275
(c)	*I_CO_*	0.120	0.165	0.244	0.289	0.352	0.430	0.413
	*I_SO_*	0.081	0.105	0.225	0.261	0.298	0.355	0.351
(d)	*I_CO_*	0.120	0.165	0.300	0.315	0.419	0.481	0.472
	*I_SO_*	0.081	0.165	0.177	0.195	0.208	0.235	0.241
